# Evaluating the tightness of proximal contact between natural tooth and adjacent implant supported prosthesis

**DOI:** 10.6026/973206300200644

**Published:** 2024-06-30

**Authors:** Madhu Ranjan, Arun Vashisht, JK Savita, Sareen Duseja, Ashish Sethi, Rajat Mohanty, Nirav H Parekh, Miral Mehta

**Affiliations:** 1 Department of Prosthodontics and Crown and Bridge, Dental Institute, RIMS, Ranchi, Jharkhand, India; 2 GimmeSmile PLLC, 2935 Tory hill lane, Sugarland Texas 77478, North America; 3 Dept of Oral Pathology & Microbiology, Rajarajeswari Dental College & Hospital, Banglore, India; 4 Department of Prosthodontics and Crown & Bridge, Narsinhbhai Patel Dental College & Hospital, Sankalchand Patel University, Visnagar, Gujarat, India; 5 Senior Consultant, Department of Dentistry, Delhi Institute of Healthcare and Research, New Delhi, India; 6 Department of Oral and Maxillofacial Surgery, Kalinga Institute of Dental Sciences, Kalinga Institute of Industrial Technology, KIIT Deemed to be University, Bhubaneswar, Odisha, India; 7 Private Dental Consultant, CT, USA; 8 Department of Pedodontics, Karnavati School of Dentistry, Karnavati University, Gandhinagar, Gujarat, India

**Keywords:** Interproximal contact, implant supported prosthesis, natural tooth

## Abstract

The changes in interproximal contact between implant supported prosthesis (ISP) and adjacent natural tooth is of interest to dentists. Hence, we evaluated the tightness of proximal contact (PCT) between adjacent natural tooth and ISP by applying a digital force gauge spanning over a period of 1.5 year with a regular follow-up of 3, 6, and 12 months.80 patients who received ISP were included in this study. In order to measure the PCT, every patient seated in the identical upright position in the dentist chair. The digital force gauge was used to take measurements for mesial PCT and distal PCT. The mesial as well as distal interproximal contacts was more tight as in case of natural tooth adjacent to other natural tooth as compared to interproximal contacts between ISP and adjacent natural tooth. It was also observed that as the time progressed there was decrease in PCT values in both categories. After 12 month follow up 30.6% cases in category 2 while 21.2% cases in category 1 showed complete loss of interproximal contact. There is significant change in proximal contact tightness in interproximal area between implant supported prosthesis and adjacent natural tooth over a period of time and necessary measures should be taken to prevent or reduce it.

## Background:

For an implant-supported prosthesis (ISP), an ideal proximal contact (PC) is crucial because it preserves the structural stability of the arch, improves masticatory effectiveness, and lowers the incidence of problems associated with tissues surrounding dental implant [1, 2-3]. The dimension and positioning of the contact region are determined by factors such as crowding of teeth, biting power, age, and tooth positioning. The contact surfaces typically have an oval contour and are located near the buccal end of the interproximal zones [4, 5-6]. According to a study, the size of the interproximal contact area shrinks while moving from posterior region of jaw toward anterior region of jaw, whether there is wear or not [7, 8-9]. They proposed that in order to prevent attrition in regions of higher biting pressure in the teeth of posterior region, bigger contact surfaces are required. In response to physiological meandering and attrition, the shape of contact areas gradually shifts from oval to kidney-shaped [10, 11-12]. Dental implants are ankylosed to bone, which prevents regular physiological phenomena like physiological movement or mesial drifting. These are present in natural teeth that are encompassed by sturdy bone along with cushioned periodontal ligament [13, 14, 15-16]. It may be among the causes of the recently reported, obvious problem of PC deterioration between the neighboring natural teeth and the implant prosthesis [17, 18-19]. One of the variables promoting the mesial displacement of teeth is an elevated level of anterior and lingual section forces as well as an excessive occlusal force exertion in the intercanine area [20, 21- 22]. Due to the ongoing eruption of neighboring teeth and possibly development of facial bone during adulthood, that affects the relative position of the teeth, an ankylosed implant also runs the risk of eventually becoming established in an infraocclusion [23, 24-25]. This risk was highlighted by a study that reported open contacts at thirty-four percent of evaluated sites. The change in the positional relationship between the implant-supported fixed prostheses (IFPs) and the adjacent natural teeth results from a dynamic oral function or the changes in other oral structures [21-24]. Therefore, it is of interest to evaluate the tightness of proximal contact (PCT) between adjacent natural tooth and ISP by applying a digital force gauge spanning over a period of 1 year with a regular follow-up of 3, 6, and 12 months.

## Materials and Methods:

This intervention study includes eighty individuals who received treatment with single first molar ISP. The age range of 18 to 50 years was applied for both both male as well as female participants in the study. The study comprised patients who had their ISP in first molar area of mandible. Included were all ISP having a adjacent natural teeth and antagonistic natural teeth in opposite arch, adjacent quadrants without any prosthesis, and restorations in proximal areas. Every single case was carefully inspected for anodontia or competitions of development of the mandible with totally erupted third molars. Following surgical extraction of their impacted third molars, the patients were then admitted to the research.

## Among the exclusion standards were:

[1] Severe gingivitis,

[2] Space between the back teeth

[3] Neighboring teeth with a >1 mobility score

[4] Adjacent teeth that have apical pathology

[5] A serious case of malocclusion

[6] Those who have their third molars erupting.

[7] People who smoke

[8] Those who are immuno-compromised

[9] Those with incapacitating illnesses

[10] Those taking drugs known to impede the healing of wounds and bones

[11] Those who exhibit parafunctional behaviors

Equipment for PCT inspection consists of a digital force gauge equipped with a metal strip that is 50 µm thick. The hospital's oral surgeons including periodontists surgically implanted dental implants in the missing teeth mandibular location for each of the eighty participants who were part of the trial. Pre-surgical examination, including radiographic assessment of the missing teeth location utilizing cone-beam computed tomography (CBCT), premedication and hemogram was performed prior to the placement of dental implant surgery. Applying a surgical kit and a physio-dispenser the dental implants were surgically placed with the proper torque and speed based on the quality (density) of the edentulous bone.

In accordance with the manufacturer's guidelines, the osteotomy was prepared by sequentially drilling with drills of diameters 2.0 mm, 2.8 mm, 3.2 mm, 3.65 mm, 4.2 mm, and 5.2 mm while receiving enough irrigation. All of the implants were inserted utilizing the open flap technique, covered by soft tissue throughout the healing period, and a program for delayed loading was established for three to four months later. Following a period of 3-4 months, the healed abutments were positioned over the implants following second-stage surgery. For approximately ten days, gingival healing was permitted around the healed abutment. The polished and completed prosthesis was inserted into the patient by introducing it over the implant, and the torque wrench was used to secure the retention screw with a torque of 20 to 30 N. The access hole was polished after being filled with composite resin. To obtain mesial and distal PCT values comparable to the first molars in the contralateral quadrant, all final prostheses were modified. Finally the prostheses glazed in the laboratory before the final cementation procedure.

Of the eighty patients, twenty had screw-retained prostheses, ten had cement-retained prostheses, and fifty had a screw-cum cement-retained prosthesis combination. They were divided into category one and category two. In each patient the quadrant that received ISP was considered as intervention category while in each patient, contralateral quadrant of same arch with no prosthesis was considered as control category. Intervention category was considered as category one while control category was category two.

Category one = intervention category (n=80).

Category two = control category (n=80).

## Measurement of proximal contact tightness:

In order to measure the PCT, every patient seated in the identical upright position in the dentist chair. The digital force gauge was used to take measurements. It consists of a metal shank bearing a hook that is attached to the digital gauge's sensor. Through perforations on the metal strip, the hook firmly grasps a 50-µm thick piece. The metal strip was placed into the digital gauge's hook, introduced interdentally from the occlusal direction, and dragged buccolingually in order to take measurements of PCT. When the strip was gradually eliminated in a bucco-lingual direction, the maximal frictional force was used to quantify the tightness of the proximal contact.

The output voltage is converted into Newton, and it could measure up to 5 N. The maximum force by pull was recorded by the digital gauge for each measurement when it was switched to peak mode. Four measurements were made at each site with the target maximum range of 5.0 N. Mesial and distal PCT values of the mandibular first molar (natural teeth) were recorded in control group.

Mesial and distal PCT values between ISP having an adjacent natural tooth were recorded in intervention group.

To avoid bias, each measurement was carried out by a single, qualified professional investigator under double blind conditions. The mean value of the four results from four measurements at a single measuring site was the outcome. Four time points were used to record contact tightness:

T0= the moment the crown was delivered

T1 = three months later

T2= six months later,

T3 = a year later

After a year, the PCT levels were statistically assessed. The contact was deemed open if there was no opposition to the buccolingual pull.

## Statistical analysis:

After being gathered, cleaned, and input into Microsoft Office Excel, the data were moved to IBM SPSS Statistics version 2.0 (IBM Corp.). It was done with an independent sample t-test. It was considered statistically significant when P < 0.05.

## Results:

The mesial PCT values was comparable in both category two (3.51±0.81) and category one (3.49± 0.86) at baseline. It showed that proximal contact tightness was comparable in both ISP and no ISP(Table 1).

The mean PCT values in category two and category one was 2.51±0.81 and 1.71± 0.86 respectively at 3 months follow up. The values reflected more tightly proximal contact in between two natural teeth as compared to ISP and natural teeth (Table 2).

The PCT observations in category one was 1.95±0.81 while it was 1.16± 0.86 in category two at 6 months follow up. The interproximal contact between ISP and adjacent natural tooth was less tight as compared to interproximal contact between natural tooth and adjacent natural tooth. The findings were significant statistically (Table 3).

The PCT values reported in category one at 12 month follow up was 1.95±0.81 while it was 0.77± 0.86 as reported in category two. The mesial as well as distal interproximal contacts was more tight as in case of natural tooth adjacent to other natural tooth as compared to interproximal contacts between ISP and adjacent natural tooth (Table 4). The findings were significant statistically. It was also observed that as the time progressed there was decrease in PCT values in both categories.After 12 month follow up 30.6% cases in category 2 while 21.2% cases in category 1 showed complete loss of interproximal contact. The complete loss of contacts was greater in ISP with adjacent natural tooth as compared to natural tooth adjacent to natural tooth.

## Discussion:

As natural teeth are surrounded by strong bone and a cushioned periodontal ligament, dental implants are ankylosed to the bone, which inhibits normal physiological phenomena like physiological movement or mesial drifting [14, 15]. It is one of the causes of the recently reported, evident issue of PC deterioration between the adjacent natural teeth and the implant prosthesis. A dynamic oral function or changes in other oral structures cause a shift in the positioning relationship between the implant-supported fixed prostheses (IFPs) and the neighboring natural teeth [18- 23]. Evaluation for this proximal contact loss consequence has received little attention in research. This study was conducted to evaluate the tightness of proximal contact (PCT) between adjacent natural tooth and ISP by applying a digital force gauge spanning over a period of 1.5 year with a regular follow-up of 3, 6, and 12 months.

In our study, the PCT values reported in category one at 12 month follow up was 1.95±0.81 while it was 0.77± 0.86 as reported in category two. The mesial as well as distal interproximal contacts was more tight as in case of natural tooth adjacent to other natural tooth as compared to interproximal contacts between ISP and adjacent natural tooth. The findings were significant statistically. It was also observed that as the time progressed there was decrease in PCT values in both categories. After 12 month follow up 30.6% cases in category 2 while 21.2% cases in category 1 showed complete loss of interproximal contact. The complete loss of contacts was greater in ISP with adjacent natural tooth as compared to natural tooth adjacent to natural tooth.

The findings of our study have some resemblance to findings of other studies showing loss of PC between ISP and natural tooth [13-20]. Some studies like our study also showed decrease in PCT between ISP and natural tooth over a period of time [11-17]. The contact surfaces are situated close to the buccal end of the interproximal zones and usually have an oval shape [12- 14]. Whether or not there is wear, a study shows that the interproximal contact area reduces as it moves from the posterior to the anterior portion of the jaw [13-19]. They suggested that larger contact surfaces are needed to minimize attrition in areas of higher biting pressure in the posterior part of the teeth. The form of contact areas eventually changes from oval to kidney-shaped due to physiological meandering and attrition [20-25].

There are some studies showing decreased tightness of PC between ISP and natural teeth as compared to PC between natural tooth and natural tooth [14-21]. According to a study, high levels of anterior and lingual section forces as well as excessive occlusal force exertion in the intercanine area are among the factors that contribute to teeth moving mesially [13-17]. An ankylosed implant also bears the risk of eventually getting established in an infraocclusion because to the continued eruption of nearby teeth and the potential development of facial bone during maturity, which alters the relative position of the teeth. According to a study, there was a danger associated with open connections at 34% of the sites that were studied [18- 24]. A dynamic oral function or modifications to other oral structures cause a change in the positioning relationship between the implant-supported fixed prostheses (IFPs) and the neighboring natural teeth [25- 26]. An optimal proximal contact (PC) is essential for an implant-supported prosthesis (ISP) since it maintains the arch's structural integrity, enhances masticatory efficacy, and reduces the likelihood of complications related to the tissues around the dental implant [11-13]. Age, biting force, tooth placement, and tooth crowding are some of the factors that affect the size and location of the contact region [14-17].

## Conclusion:

Data shows that there is significant change in proximal contact tightness in interproximal area between implant supported prosthesis and adjacent natural tooth over a period of time and necessary measures should be taken to prevent or reduce it.

## Figures and Tables

**Table 1:** Mean mesial and distal PCT values at time of placement of ISP

	**Category Two**	**Category One**
Mesial PCT	3.17± 0.84	3.21 ±0.92
Distal PCT	3.83±0.82	3.76±0.84
Mean PCT	3.51±0.81	3.49± 0.86
P value	1.478	

**Table 2:** Mean mesial and distal PCT values at 3 months follow up

	**Category Two**	**Category One**
Mesial PCT	2.17± 0.84	1.67 ±0.92
Distal PCT	2.98±0.82	1.85±0.84
Mean PCT	2.51±0.81	1.71± 0.86
P value	0.001	

**Table 3:** Mean mesial and distal PCT values at 6 months follow up

	**Category one**	**Category two**
Mesial PCT	1.87± 0.84	1.07 ±0.92
Distal PCT	2.08±0.82	1.25±0.84
Mean PCT	1.95±0.81	1.16± 0.86
P value	0.001	

**Table 4:** Mean mesial and distal PCT values at 12 months follow up

	**Category one**	**Category two**
Mesial PCT	1.27± 0.84	0.79 ±0.92
Distal PCT	1.07±0.82	0.65±0.84
Mean PCT	1.95±0.81	0.77± 0.86
P value	0.001	
